# Knowledge of chronic spontaneous urticaria management among Italian general practitioners: Baseline findings from the BRIDGE study^☆^

**DOI:** 10.1016/j.waojou.2026.101404

**Published:** 2026-05-29

**Authors:** Piergiacomo Calzavara-Pinton, Enrico Iemoli, Pallavi Saraswat, Nadine Chapman-Rothe, Ornella Bonavita, Tara Raftery, Vinodh Nallasamy, Mahrukh Zahid, Viviane Sprecher, Abigail Herbst, Bridget Gaglio, Fabio Speranza, Patrick Daniele, Riccardo Asero

**Affiliations:** a Dermatology Department, University of Brescia, Brescia, Italy; b Allergy and Clinical Immunology Unit, ASST FBF-Sacco, Milan, Italy; c Novartis Healthcare Pvt Ltd, Hyderabad, India; d Novartis Pharma AG, Basel, Switzerland; e Novartis Farma, SpA, Milan, Italy; f Novartis Ireland Ltd, Dublin, Ireland; g PPD Evidera Patient-Centered Research, Thermo Fisher Scientific, Wilmington, NC, USA; h PPD Evidera Patient-Centered Research, Thermo Fisher Scientific, London, UK; i PPD Evidera Patient-Centered Research, Thermo Fisher Scientific, Vancouver, Canada; j Clinica San Carlo, Ambulatorio di Allergologia, 20037, Paderno Dugnano, Italy

**Keywords:** Chronic spontaneous urticaria, Effectiveness-implementation study, Patient-reported outcomes, CSU management, General practitioners

## Abstract

**Introduction:**

Patients with chronic spontaneous urticaria (CSU) often experience diagnostic delays and uncontrolled disease, highlighting the need to improve CSU care. The BRIDGE study, a hybrid type 1 effectiveness-implementation study, evaluated the effectiveness and feasibility of an urticaria care package (UCP) to increase patient-reported outcome (PRO) use in clinical practice and improve CSU care among general practitioners (GPs). Two surveys were conducted, 1 before and 1 after UCP implementation. This report focuses on pre-implementation data regarding the management and knowledge of CSU among GPs in Italy.

**Methods:**

Eligible GPs were from Lombardy, an administrative region of Italy, who had been in clinical practice for 3–30 years and had access to electronic medical records. GPs completed a self-administered, web-based survey, prior to UCP implementation. Survey questions included the following topics: awareness and knowledge of CSU, use of CSU-specific PROs or other PROs, attitudes towards UCP adoption, implementation climate, and key factors influencing UCP adoption. The analysis included GPs who completed ≥1 item on the pre-implementation survey.

**Results:**

The analysis included 95 GPs (mean age: 37.7 years; female: 50.5%; mean time practicing medicine: 10.1 years). On average over the previous 6 months, GPs managed 3.6 patients with suspected CSU, diagnosed 2.3 patients with CSU, treated 2.1 patients for CSU, and referred 3.0 patients with suspected CSU to a dermatologist or allergist. GPs' primary reasons for referral were seeking confirmation of CSU diagnosis (61.0%), seeking the specialist's opinion (51.2%), and not feeling confident in diagnosing or treating patients with CSU (42.7%). Most GPs had little to no knowledge of CSU (60.0%) or were unaware of the existence of CSU guidelines (61.1%). Among those who were aware of the guidelines, 35.1% applied them on a regular basis. Only 14.7% of GPs reported any use of PROs, and none used PROs with patients with CSU in the past 6 months. Lack of time hindered GPs from discussing PROs with patients all the time (28.6%), most of the time (42.9%), or sometimes (28.6%). Most GPs reported no or limited educational support on evidence-based practices from their clinics, and indicated the importance of further education on CSU diagnosis and management (72.6% important; 7.4% very important) and further training on PRO use (61.1% important; 8.4% very important) for enhancing CSU management.

**Conclusion:**

Pre-implementation data revealed substantial knowledge gaps in CSU management among GPs in Lombardy, supporting the need for additional training to improve CSU management.

## Introduction

Chronic spontaneous urticaria (CSU) is characterized by the occurrence of relapsing/remitting eruptions of swollen and itchy wheals (hives) and/or angioedema persisting for over 6 weeks, typically in the absence of a definite external trigger.^1^^,^^2^ Globally, CSU affects approximately 0.5–1% of the population, depending on the region.^3^^,^^4^ In Italy, the sex- and age-adjusted prevalence of CSU is about 1.3%.^5^

The average disease duration in CSU is approximately 2–5 years;^6^ however, symptoms may persist much longer in some patients.^3^ Based on a review of studies in patients with CSU, the weighted average cumulative remission rate is approximately 17% within 1 year, 45% within 5 years, and 73% within 10 years.^7^ CSU interferes with work, sleep, and daily activities of patients, impairing their health-related quality of life (HRQoL).^3^^,^^8^^,^^9^ Additionally, the burden of CSU extends beyond physical well-being, substantially affecting the emotional health of patients.^10^^,^^11^ Real-world data showed that approximately 30%–50% of Italian patients with CSU experienced moderate to extremely high disease impact on HRQoL,^5^^,^^12^^,^^13^ and more than 70% of the patients experienced mild to severe anxiety and depression.^5^

Despite the significant disease burden, patients with CSU often experience delays in diagnosis and initiation of appropriate treatment.^9^^,^^14^ In many cases, patients consult with at least 2 physicians before receiving a diagnosis of CSU.^13^^,^^15^^,^^16^ Delays in making a diagnosis and previous physicians not being able to identify effective therapy are common reasons for switching physicians.^13^ Conversely, many patients undergo laboratory testing, dietary modifications, and therapies that are outside of clinical practice guidelines,^1^^,^^10^^,^^17^ which can potentially delay appropriate treatment. Delays in treatment escalation^10^^,^^16^ can further prolong the disease journey. Moreover, some patients report that their disease severity is often underestimated by their primary care physicians or specialists.^11^ These findings highlight the unmet needs in CSU care.

Patient-reported outcomes (PROs) can measure aspects of a disease that matter most to patients but may not be captured by standard clinical outcomes.^18^ Current guidelines for CSU management recommend the use of PROs in clinical practice to monitor disease activity, impact of disease on HRQoL, and treatment response, which can facilitate timely adjustment of the treatment plan when needed.^1^ Multiple PRO measures, such as the weekly Urticaria Activity Score, Urticaria Control Test, Chronic Urticaria Quality of Life Questionnaire, and Dermatology Life Quality Index, are available to assess disease activity, sleep quality, and HRQoL in CSU from the patient's perspective.^19^, ^20^, ^21^

General practitioners (GPs) are often the initial point of contact for patients experiencing CSU symptoms and therefore play an important role in CSU care. However, the knowledge and application of these PRO measures among GPs have not been previously described. Understanding the knowledge gaps in current management of CSU is essential for guiding targeted education and training strategies intended to improve disease management in primary care.

The BRIDGE study was a hybrid type 1 effectiveness-implementation study with a pre-post design to evaluate the effectiveness and feasibility of an educational urticaria care package (UCP) aimed at increasing PRO use in clinical practice and improving CSU management. Two surveys informed by implementation science methodology were conducted, 1 before and 1 after the implementation of the UCP, to assess changes in PRO use and CSU management, particularly changes in the diagnosis and referral of suspected CSU cases. This paper presents the pre-implementation survey results, providing insights into the current use of PROs among GPs in the diagnosis and management of CSU.

## Methods

### Study design

BRIDGE was a hybrid type 1 effectiveness-implementation study. This allowed for the simultaneous evaluation of both the effectiveness and implementation of the UCP, with a primary focus on effectiveness outcomes.^22^^,^^23^ The study consisted of 4 phases: contextual analysis; pre-implementation; implementation; and post-implementation. The contextual analysis identified key concepts related to diagnosis, referral, and treatment escalation in CSU treatment pathway^24^ and informed the development of the UCP and the pre- and post-implementation surveys. The resulting UCP contained training and educational materials on disease background and PRO use in CSU, as well as guidance on referral networks. The Consolidated Framework for Implementation Research (CFIR) Version 1^25^, ^26^, ^27^ was used to guide the examination of factors that influence the implementation of the UCP. Italy was selected as the pilot country for UCP implementation. The UCP will be disseminated and implemented more widely within Italy and in additional countries dependent upon the results of the pilot.

### Study population

GPs and specialists in Lombardy, a region in northern Italy, were recruited by a local clinical research organization engaging the *Scuola Italiana di Formazione e Ricerca in Medicina di Famiglia* (SIFMed; a GP association). A feasibility questionnaire was sent to the SIFMed email distribution list. GPs were eligible if they had been in clinical practice for 3–30 years and had access to electronic medical records.

### Ethics statement

The study was conducted in accordance with the principles of the Declaration of Helsinki. Based on local regulation and the guidelines from the *Agenzia Italiana del Farmaco* (AIFA; Italian Medicines Agency) for the conduction of non-interventional studies, approval by an ethics review board was not needed for this study as no patients were involved.

### Data collection

Participants took part in a self-administered, web-based survey, prior to the implementation of the UCP. The 30-min survey, consisting of 63 questions, was conducted between May 2024 and July 2024. The survey covered constructs identified from the contextual analysis and the domains and measures aligned with the CFIR. Demographic and professional information of participants, including age, gender, and years in medical practice, were collected. Participants were also asked to answer questions pertaining to the following topics: awareness and knowledge of CSU, use of generic and CSU-specific PROs, attitudes towards adoption of the UCP, implementation climate, and key factors influencing adoption of the UCP. To ensure the quality and integrity of the study results, the web-based data collection system included automatic data validation checks. Remote manual data quality reviews were performed by the data analysts.

### Sample size and statistical analysis

It was estimated that a sample of 95 GPs would provide 81.1% power to detect an odds ratio of 2.60 using a two-sided McNemar test at a significance level of 0.05. Considering approximately 5% of invalid assessments, including lost to follow-up, the target sample size was 100 GPs to allow for assessment of change in PRO use.

The analysis included data for GPs who completed at least 1 item on the pre-implementation survey. Descriptive analysis was conducted to understand current PRO use and CSU management among GPs prior to UCP implementation. Continuous variables were summarized using simple descriptive statistics (mean, standard deviation, median, quartiles, minimum, and maximum). Categorical variables were summarized with frequencies and proportions. No prespecified hypotheses were tested. No missing data were imputed or prorated. Participants who did not respond to a particular question or were not presented with it due to skip logic were not included in the denominator for that specific question. SAS version 9.4 (SAS Institute Inc., Cary, NC, USA) was used for all analyses.

## Results

### Participant disposition and characteristics

One-hundred eleven GPs were invited to participate in the baseline survey, and 96 agreed to participate. Ninety-five GPs (85.6%) completed the survey (Supplemental Fig. 1). The mean age of GPs was 37.7 years (standard deviation 7.9), and 50.5% were female (Table 1). GPs had been practicing medicine for an average of 10.1 years (standard deviation 7.3). Most GPs (80.0%) worked in a single practice that collaborates with a group/association. Most practices were located in urban areas (56.8%), with smaller numbers of practices in suburban areas (32.6%) and rural areas (8.4%).

**Table 1:** Participant characteristics.

Demographic and practice characteristics	Participants (N = 95)
**Age, years**	n = 93
Mean (SD)	37.7 (7.9)
Median (min, max)	36 (28, 68)
**Gender, n (%)**	n = 95
Male	47 (49.5)
Female	48 (50.5)
**Years of experience practicing medicine**	n = 95
Mean (SD)	10.1 (7.3)
Median (min, max)	8 (3, 30)
**Practice type, n (%)**	n = 95
Individual practice	9 (9.5)
Single practice in collaboration with group/association	76 (80.0)
Other	10 (10.5)
**Practice location, n (%)**	n = 95
Urban area	54 (56.8)
Suburban area	31 (32.6)
Rural area	8 (8.4)
Other^a^	2 (2.1)

Max: maximum; min: minimum; SD: standard deviation.

a Two participants each selected two practice locations and were categorized as “other” to facilitate interpretation of the results.

### Experience with CSU care

On average, GPs had suspected 3.6 patients of having CSU, diagnosed 2.3 patients with CSU, and treated 2.1 patients for CSU over the previous 6 months (Table 2). GPs reported referring an average of 3.0 patients with a suspected diagnosis of CSU to a dermatologist or allergist in the previous 6 months. Among GPs who indicated that they had suspected at least 1 patient of having CSU in the past 6 months, the median (range) time between referral and a specialist appointment for their patients was 10 (1–30) days for the private practice setting and 100 (9–450) days for the national health service (*Sistema Sanitario Nazionale*) (Table 2). Among GPs who indicated they had diagnosed at least 1 patient with CSU in the previous 6 months, the median (range) time between patients experiencing CSU symptoms and receiving a diagnosis was 3 (0–20) months (Table 2).

**Table 2:** Management of patients with CSU.

CSU management characteristics	Participants (N = 95)
**Number of patients with suspected CSU in the past 6 months**	n = 95
Mean (SD)	3.6 (4.4)
Median (min, max)	2 (0, 30)
**Number of patients diagnosed with CSU in the past 6 months**^a^	n = 85
Mean (SD)	2.3 (2.8)
Median (min, max)	2 (0, 15)
**Number of patients with CSU treated in the past 6 months**^a^	n = 85
Mean (SD)	2.1 (2.5)
Median (min, max)	2 (0, 15)
**Number of patients with suspected CSU referred to a dermatologist or allergist**^a^	n = 85
Mean (SD)	3.0 (2.9)
Median (min, max)	2 (0, 20)
**Time between referral and first appointment with a specialist, days**^b^	
Referral to a private specialist	n = 77
Mean (SD)	12.5 (8.0)
Median (min, max)	10 (1, 30)
Referral to *Sistema Sanitario Nazionale*^c^	n = 81
Mean (SD)	136.7 (100.3)
Median (min, max)	100 (9, 450)
**Time between patients experiencing CSU symptoms and receiving a diagnosis, months**^d^	n = 71
Mean (SD)	4.4 (3.8)
Median (min, max)^e^	3 (0, 20)

CSU: chronic spontaneous urticaria; max: maximum; min: minimum; SD: standard deviation.

a Applicable to participants who indicated that they had suspected ≥1 patient of having CSU in the past 6 months.

b Applicable to participants who indicated that they had referred ≥1 patient with a suspected diagnosis of CSU to a dermatologist or allergist in the past 6 months.

c Italy's national health service.

d Applicable to participants who indicated that they had diagnosed ≥1 patient with CSU in the past 6 months.

e The first and third quartiles of the time between patients experiencing CSU symptoms and receiving a diagnosis were 2 and 6 months, respectively.

Among the 82 GPs who referred at least 1 patient with suspected CSU to a specialist in the previous 6 months, seeking “confirmation of CSU diagnosis” (n = 50/82, 61.0%) or “a specialist's opinion” (n = 42/82, 51.2%) were most frequently cited as the reasons for referral to a dermatologist or allergist. Feeling unconfident in diagnosing or treating patients with CSU was cited as the reason for referral by 35 out of 82 GPs (42.7%). Some GPs referred patients to a specialist because they did not consider themselves a CSU expert (n = 22/82, 26.8%), the patient's case was complex and required specialist care (n = 20/82, 24.4%), they believed patients with CSU should be managed by dermatologists or allergists (n = 3/82, 3.7%), the patient wanted a second opinion (n = 3/82, 3.7%), they were not authorized to prescribe the necessary treatment in their practice (n = 2/82, 2.4%), or for other unspecified reasons (n = 5/82, 6.1%).

Among the 71 GPs who had diagnosed at least 1 patient with CSU in the previous 6 months, peer-reviewed literature (n = 39/71, 54.9%), colleagues (n = 33/71, 46.5%), or CSU clinical practice guidelines (n = 24/71, 33.8%) were the main resources GPs consulted when making the diagnosis. A small proportion of participants consulted with a specialist virtually (n = 5/71, 7.0%) or consulted CSU-specific point of care tools (n = 2/71, 2.8%). However, 15.5% (n = 11/71) of GPs did not consult any resources to make a CSU diagnosis.

### Knowledge of CSU and CSU guidelines

Most GPs (60.0%) had little to no knowledge of CSU (Table 3). Only about one-fifth of GPs (18.9%) were knowledgeable about CSU and had managed patients with the disease. Most GPs (61.1%) were not aware of the existence of specific guidelines on the management and treatment of CSU. Of the 37 GPs who indicated that they were aware of CSU guidelines, 13 (35.1%) applied them regularly, 22 (59.5%) applied them but not on a regular basis, and 2 (5.4%) did not apply them at all. Those who had used CSU guidelines reported referring to a range of different guidelines in practice.

**Table 3:** Knowledge of CSU and its management guidelines among GPs.

Survey items	Participants (N = 95), n (%)
**Knowledge of management of CSU**	n = 95
I have knowledge and experience managing patients with CSU	18 (18.9)
I have knowledge of the condition but have not managed a patient with CSU	20 (21.1)
I have little knowledge of CSU	55 (57.9)
I have no knowledge of CSU	2 (2.1)
**Awareness of existence of CSU guidelines**	n = 95
Yes	37 (38.9)
No	58 (61.1)
**Frequency of CSU guideline use**^a^	n = 37
I apply the guidelines regularly	13 (35.1)
I apply the guidelines, though not regularly	22 (59.5)
I don't apply the guidelines	2 (5.4)

CSU: chronic spontaneous urticaria; GP: general practitioner.

a Applicable to participants who indicated they were aware of existence of specific guidelines on the management and treatment of patients affected by CSU.

### Knowledge of PROs for CSU

Of all 95 GPs, 48 (50.5%) had no professional experience using PROs in their routine clinical care of any patients, and 33 (34.7%) were aware of PRO measures but had never used them during their routine clinical care. Only 14 GPs (14.7%) had experience of using PROs, although none of them had used PROs with patients with CSU in the previous 6 months. GPs indicated that lack of time was a barrier to them discussing PROs with patients most of the time (n = 6/14, 42.9%), all the time (n = 4/14, 28.6%), or sometimes (n = 4/14, 28.6%).

**Fig. 1:**
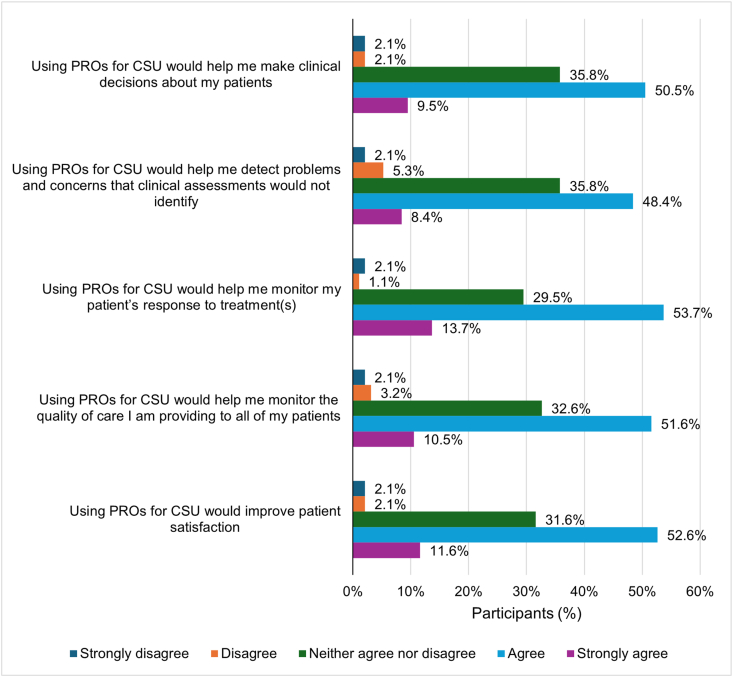
**Benefits of PROs perceived by GPs(N=95).** CSU: chronic spontaneous urticaria; GP: general practitioner, PRO: patient-reported outcome

Most GPs agreed or strongly agreed that using PROs for CSU would help them make clinical decisions about their patients (50.5% agreed; 9.5% strongly agreed), detect problems and concerns that would not be identified by clinical assessment (48.4% agreed; 8.4% strongly agreed), monitor their patients’ treatment responses (53.7% agreed; 13.7% strongly agreed) or the quality of care they were providing (51.6% agreed; 10.5% strongly agreed), or improve patient satisfaction (52.6% agreed; 11.6% strongly agreed) (Fig. 1).

### Perspectives on improvement of CSU knowledge and management

**Fig. 2:**
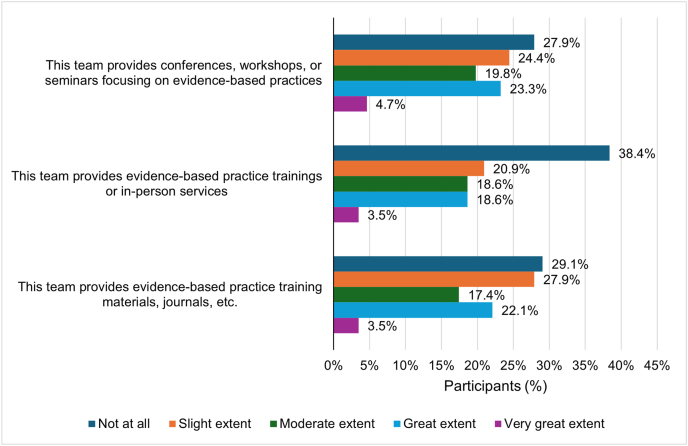
**Educational support provided by the clinical practice team of the GP on evidence-based practice (N = 86).** Questions were asked only of participants who did not have an individual practice. The team refers to people other than the GP in the work group, including physicians in the same or different specialty, nurses, or others in the GP's office. Participants selected the number indicating the extent to which they agreed with each item using the following scale: 0 – not at all; 1 – slight extent; 2 – moderate extent; 3 – great extent; 4 – very great extent. GP: general practitioner

More than half of GPs reported no or only limited availability in their clinics of workshops/seminars focusing on evidence-based practices (EBPs) and EBP training materials/journals (Fig. 2). Specifically, for most GPs, their team provided no or limited support including EBP-focused conferences, workshops, or seminars (27.9% not at all; 24.4% slight extent), EBP trainings or in-person services (38.4% not at all; 20.9% slight extent), and EBP training materials, journals, or other related materials (29.1% not at all; 27.9% slight extent).

Over half of GPs (55.8%) perceived changes for CSU care in clinical practice to be of medium priority, and 12.6% perceived it to be of high priority. Improving CSU care relative to other initiatives were considered of medium priority by 55.8% of GPs and high priority by 23.2% (Table 4). Further educational support on the diagnosis and management of CSU was perceived as important by 72.6% of GPs and very important by 7.4%, while further training on PRO use was perceived as important by 61.1% and very important by 8.4% (Table 4). Inclusion of documents and job aids (72.6%), web/mobile application-based training (68.4%), and connecting with professionals in other clinics (56.8%) were the modalities for training preferred by most GPs (Table 4).

**Table 4:** GPs’ perspectives on CSU management in Italy.

Survey items	Participants (N = 95), n (%)
**Priority for change for CSU care in clinical practice**	n = 95
Not a priority	2 (2.1)
Low priority	27 (28.4)
Medium priority	53 (55.8)
High priority	12 (12.6)
Essential	1 (1.1)
**Priority for improving CSU care relative to other initiatives**	n = 95
Not a priority	0 (0.0)
Low priority	19 (20.0)
Medium priority	53 (55.8)
High priority	22 (23.2)
Essential	1 (1.1)
**Importance of further training on diagnosis and management of CSU**	n = 95
Very unimportant	0 (0.0)
Unimportant	3 (3.2)
Neutral	16 (16.8)
Important	69 (72.6)
Very important	7 (7.4)
**Importance of further training on the use of PROs for CSU**	n = 95
Very unimportant	0 (0.0)
Unimportant	5 (5.3)
Neutral	24 (25.3)
Important	58 (61.1)
Very important	8 (8.4)
**Training preferences**^a^	n = 95
Is web- or mobile application-based	65 (68.4)
Includes documents and job aids	69 (72.6)
Is completed with my colleagues	21 (22.1)
Is completed on my own	24 (25.3)
Presents scenarios to convey information	25 (26.3)
Connects me with professionals in other clinics	54 (56.8)
Incorporates the patient's voice	5 (5.3)

CSU: chronic spontaneous urticaria; GP: general partitioner; PRO: patient-reported outcomes.

a Responses are not mutually exclusive.

## Discussion

This analysis of the pre-implementation data from the BRIDGE study provided insights into the practices of GPs in Lombardy when managing CSU, their awareness and use of PROs in CSU care, and the barriers to CSU diagnosis and management. GPs mainly managed CSU in collaboration with specialists and had limited knowledge of CSU and its management. A substantial proportion of GPs reported lacking confidence in independently diagnosing or treating CSU, leading them to refer patients to specialists. PROs were largely underused, with only 14.7% of GPs reporting any use of PROs and none reporting PRO use for patients with CSU in the past 6 months. Although GPs acknowledged various benefits of using PROs in CSU management, lack of time often prevented them from using PROs in the clinic. GPs recognized the importance of education and training on PRO use and CSU management but indicated that they lacked sufficient support in these areas.

Management of CSU is challenging given the absence of a definite trigger. Patients experiencing CSU symptoms often first seek care from GPs. However, knowledge gaps in CSU management among GPs can cause significant delays in diagnosis and treatment. The current analysis revealed a median time of 3 months from CSU symptom onset to diagnosis, comparable to that in a previous survey of CSU specialists in Italy (median 4 months).^13^ For some patients of the surveyed GPs in the current analysis, it may take more than a year to receive a diagnosis of CSU after referral, depending on the practice setting of the specialist. This is in line with the findings from the Urticaria Voices study conducted among CSU-treating physicians (dermatologist and allergists) and patients with CSU from 7 countries (United States, Canada, United Kingdom, Germany, France, Italy, and Japan). In that study, physicians in Italy reported an average 1.2-year delay in CSU diagnosis since symptom onset,^16^ whereas the delay was estimated to be 2 years from the perspective of patients in Italy.^28^ These findings underscore the critical need to optimize CSU care in Italy.

Most GPs in the current study did not apply guidelines regularly in managing CSU, even those who were aware of them. A similar finding was also reported from the above-mentioned survey of Italian specialists, which found that only 27% of the respondents used CSU guidelines regularly in their routine practice.^13^ In the Urticaria Voices study, globally, approximately 30% of the physicians reported not adhering to any guidelines.^16^ Additionally, patients with CSU in that study also reported taking treatments not included in the international guidelines or not receiving treatment escalation when their disease was inadequately controlled.^10^ These results point to a clear need for improving guideline adherence.

GPs in the current study agreed that educational support on CSU management and PRO use were important to enhance CSU care. These findings are consistent with a previous survey among primary healthcare professionals for allergic conditions across 63 countries. In that study, 65% of respondents reported inadequate knowledge of urticaria, and 75% indicated educational needs for this condition.^29^

Taken together, the pre-implementation results from the BRIDGE study highlight an unmet need for additional education and training in CSU-related PROs and guidelines among GPs in Italy. These results are particularly relevant given the recent regulatory updates in the Lombardy region, which have simplified the prescribing pathway for omalizumab, a biologic agent for the treatment of CSU, by allowing GPs to issue subsequent prescriptions following the initial prescription by a specialist.^30^^,^^31^ This change in practice marks a step forward for improving the quality of life for patients with CSU by making access to treatment easier and more efficient.^30^ Moreover, the treatment landscape of CSU is rapidly expanding, with newer medications and therapeutic options anticipated to become available.^32^^,^^33^ A better knowledge of CSU and its management becomes more important and urgent among GPs. Adoption of the UCP is expected to help address the current knowledge gaps. Post-implementation data collection for the BRIDGE study has been completed, and upcoming analyses will determine the effectiveness of the UCP in increasing PRO use and improving the patient journey in clinical practice.

The study has a few limitations. As with all survey studies, data in the current study were self-reported, which may have introduced recall or response biases. While such biases may not be entirely avoidable, the implementation of consistency checks and data validation procedures may have helped ensure response accuracy and strengthen data quality, although the level of remaining bias is unknown. The study is also limited by the non-random sampling approach. The study population may not be representative of the broader target population, limiting the generalizability of our findings. Moreover, participating GPs were recruited from a single region in Italy and may not reflect the experiences and practices of all GPs who provide CSU care across the country.

## Conclusion

The pre-implementation analysis from the BRIDGE study identified considerable gaps in knowledge of optimal CSU management among GPs in the Lombardy region of Italy. PROs are infrequently used in routine clinical practice, with lack of time cited as a principal barrier, indicating missed opportunities to enhance symptom monitoring and treatment response. These findings underscore the need for targeted training and implementation support to strengthen CSU management in Italian primary care.

## Data availability

The survey questions and the datasets generated during and/or analyzed during the current study will be provided upon reasonable request.

## Author contributions

Conceptualization: OB. Methodology: BG, TR, NCR, OB, VN, PS, MZ, PD. Validation: VN, PS. Formal analysis: TR, NCR, OB, VN, PS, VS, PD. Investigation: BG; FS, AH, TR, NCR, OB, RA, EI, PCP, PS. Resources: OB, RA, EI, PCP. Data curation: BG, FS, AH, VN, PD. Writing - original draft: BG, FS, AH. Writing - review & editing: TR, NCR, OB, RA, EI, PCP, VN, PS, VS, MZ, PD, AH, BG, FS. Visualization: RA, PCP. Supervision: BG, TR, NCR, OB, PS, VS, MZ. Project administration: BG, AH, NCR, TR, OB, PS, VS. Funding acquisition: TR, NCR, OB.

## Ethics statement

The study was conducted in accordance with the principles of the Declaration of Helsinki. Based on local regulation and the guidelines from the *Agenzia Italiana del Farmaco* (AIFA; Italian Medicines Agency) for conducting non-interventional studies, approval by an ethics review board was not needed for this study as no patients were involved.

## Declaration of generative AI and AI-assisted technologies in the writing process

Nothing to disclose.

## Funding

This study was funded by Novartis Pharma AG.

## Declaration of competing interest

**Pallavi Saraswat** is an employee of Novartis Healthcare Pvt. Ltd and holds shares of Novartis Pharma AG. **Nadine Chapman-Rothe**, **Tara Raftery**, **Mahrukh Zahid**, and **Viviane Sprecher** are employees of Novartis Pharma AG and hold shares of Novartis Pharma AG. **Ornella Bonavita** is an employee of Novartis Farma. **Vinodh Nallasamy** is an employee of Novartis Healthcare Pvt. Ltd. **Abigail Herbst** and **Fabio Speranza** are employees of Thermo Fisher Scientific, which received funding for supporting study activities. **Bridget Gaglio** and **Patrick Daniele** are employees and shareholders of Thermo Fisher Scientific, which received funding for supporting study activities. **Piergiacomo Calzavara-Pinton**, **Enrico Iemoli**, and **Riccardo Asero** declare no conflicts of interest regarding this study.
