# Supplementary figures and images for: Knowledge of chronic spontaneous urticaria management among Italian general practitioners: Baseline findings from the BRIDGE study
Source: World Allergy Organ J. 2026 May 29;19(7):101404. doi: 10.1016/j.waojou.2026.101404 (PMC13241996; doi:10.1016/j.waojou.2026.101404)

## APPENDIX A. SUPPLEMENTARY DATA

**Supplemental Figure 1. Participant disposition.**

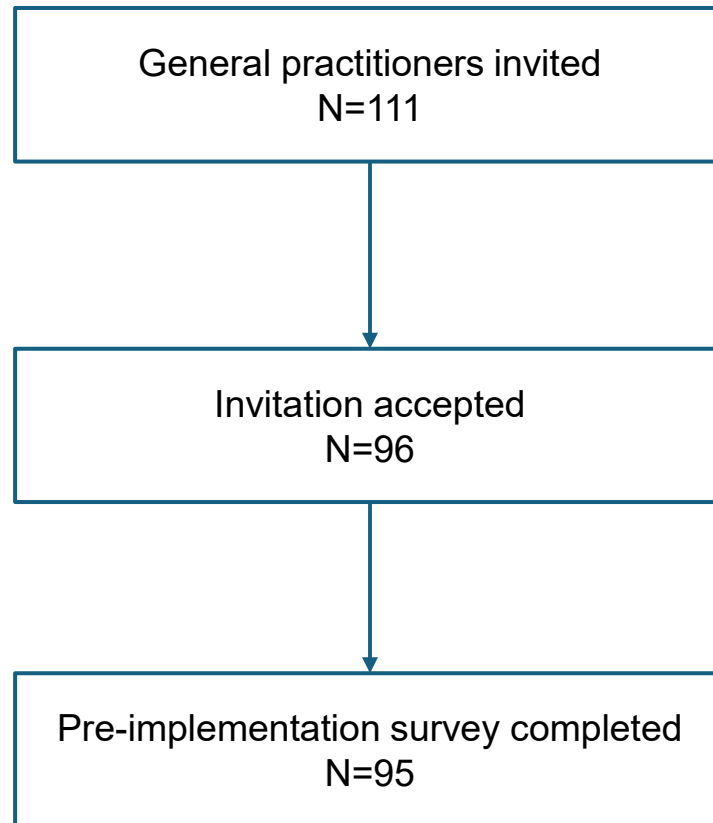

Supplement: Multimedia component 1 [file mmc1.pdf]
